# The Distribution and Fracture Patterns of Mandibular Fractures Due to Traffic Accidents: A Retrospective Study

**DOI:** 10.3390/diagnostics16081172

**Published:** 2026-04-15

**Authors:** Ömer Turan, İsmail Altın

**Affiliations:** Department of Forensic Medicine, Faculty of Medicine, Istanbul Medeniyet University, Istanbul 34722, Türkiye; dromerturan@gmail.com

**Keywords:** mandibular fractures, traffic accidents, maxillofacial injuries, fracture localization, forensic medicine

## Abstract

**Background**: Mandibular fractures constitute a significant proportion of maxillofacial trauma resulting from traffic accidents and present valuable information about the severity of the trauma mechanism. The aim of this study was to evaluate the demographic characteristics, fracture patterns, and accompanying injuries of mandibular fractures resulting from traffic accidents. **Methods**: A retrospective examination was made of 94 patients who presented for forensic medicine evaluation following a traffic accident between 1 January 2019 and 31 December 2024 and were determined with mandibular fracture. The demographic data, accident characteristics, localization of the mandibular fracture, number of fractures, displacement status, and accompanying injuries were analyzed. **Results**: The analyzed cases comprised 68.1% males and 31.9% females, with a mean age of 29.27 ± 14.34 years. The mandibular fractures were displaced in 52.1% of cases, and closed in 98.9%. The fracture regions were determined to most often be the ramus (32.9%) and the condyle (32.9%). A single fracture was present in 54.9% of cases and multiple fractures in 45.1%. A significant correlation was seen between ramus fractures and male gender, driver status, and concomitant systemic injuries, whereas no significant relationship was found between some fracture types and the demographic and accident-related variables. **Conclusions**: Mandibular fractures resulting from traffic accidents may represent relatively high-energy trauma mechanisms, and certain fracture patterns may occur together with multiple and systemic injuries. The localization and characteristics of mandibular fractures present important clues about the biomechanics of the trauma and a holistic approach is required in the forensic medicine evaluation.

## 1. Introduction

Maxillofacial traumas constitute a significant trauma group, as they have a severe effect on the quality of life of the patient because of functional and esthetic outcomes, and can lead to long-term morbidity [1,2,3]. As the mandible is located in the anterior–inferior section of the facial skeleton, with limited soft tissue support, and it is directly exposed to external traumatic forces, it is one of the most frequently fractured bones in maxillofacial trauma [1,2].

Although the mandible can be fractured at a similar degree to the zygoma, but with less energy required than for the frontal bone, it has been reported that approximately four-fold more force is required to be able to fracture the maxilla [4,5]. This may suggest that the mandible is particularly vulnerable in higher-energy trauma mechanisms [2].

**Figure 1:**
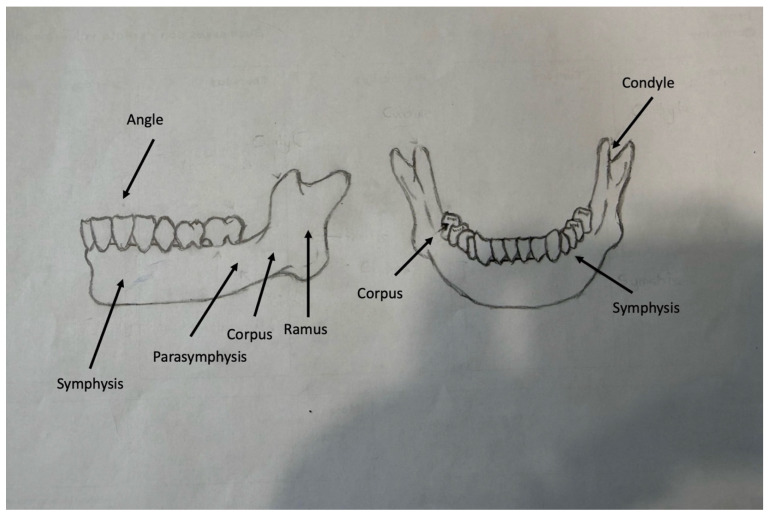
Schematic illustration of mandibular anatomic regions and fracture localization zones (lateral and inferior views).

The anatomic distribution of mandibular fractures is not homogenous, and may be concentrated in different zones depending on the severity, direction, and mechanism of the trauma. It has been reported in the literature that the posterior mandible segments (especially the condyle and ramus) are more often affected in high-energy traumas, whereas direct impacts can lead to fractures in the corpus, angle, and parasymphysis regions (Figure 1). This variable distribution pattern is closely related to the direction and magnitude of the force transferred to the mandible during the trauma [1,2,6,7,8].

Although the etiology of mandibular fractures varies between countries and societies, traffic accidents emerge as one of the most significant causes on a global scale [1,2,3]. Previous studies have shown that traffic accidents are the leading cause of mandibular fractures [1,2,9,10].

Many mandibular fractures associated with traffic accidents may be related to relatively higher-energy trauma mechanisms, and are usually not in the form of isolated lesions but are seen together with other facial bone and head–neck injuries [11,12]. In an analysis based on the German Trauma Register (DGU^®^), maxillofacial injury was determined in more than 20% of patients who had undergone traffic accidents with severe trauma, and it was reported that more than 60% of these cases had an accompanying traumatic brain injury [11].

Mandibular fractures due to traffic accidents should be evaluated not as a localized facial injury but as findings presenting important forensic medicine markers related to the severity and mechanism of the trauma [11,12]. The localization of a mandibular fracture, the number of fractures, and concomitant facial, cranial, and other system injuries provide indirect but valuable information about whether or not the accident was high-energy, the direction of the impact, and the location of the individual in the accident [7,13]. Therefore, in the forensic medicine examination of mandibular fractures resulting from traffic accidents, the presence of the fracture should not be evaluated alone, but with a holistic approach that is compatible with the mechanism of the event and the relationship with the accompanying injuries [11].

The aim of this study was to examine the demographic characteristics of patients with mandibular fracture who presented at the Forensic Medicine Department of Istanbul Medeniyet University Medical Faculty after a traffic accident, and to determine the anatomic localizations of the fractures and the fracture patterns. It was also aimed to evaluate the effects of accident-related factors (vehicle type, localization of the injured person in the accident, direction of impact) on the formation and localization of mandibular fractures, and to analyze the relationships between mandibular fractures and the accompanying facial, cranial, and other system injuries.

## 2. Materials and Methods

The study population consisted of traffic accident cases that presented at the Forensic Medicine Unit of Istanbul Medeniyet University Medical Faculty for medico-legal evaluation within the regional catchment area of the institution. During the study period, a total of 12,199 forensic medicine evaluations related to traffic accidents were retrospectively screened. Of these cases, 1141 patients were identified with documented facial trauma. Radiological records were reviewed and 105 cases with mandibular fractures were identified. After applying the exclusion criteria, 11 cases were excluded. Consequently, 94 patients with radiologically confirmed mandibular fractures were included in the final analysis.

The cases included were those determined with mandibular fracture and radiological images available (panoramic radiograph and/or computed tomography). The records of cases with no history of traffic accident, no mandibular fracture determined, or no available radiological images were excluded from the study. For cases with repeated presentations, a single record was included in the analysis. Patients were also excluded from the study if the mandibular fracture was associated with pathological fractures (tumor, osteomyelitis, etc.) or with a trauma mechanism other than a traffic accident (fall, assault, firearms injury, etc.). In some cases, radiological images were available but the anatomic localization of the fracture could not be clearly distinguished, so these cases were excluded. The cases included in the study were only those with variables that could be evaluated from the records and images and reliably determined for the analyses of fracture localization and number of fractures.

The data of the cases were retrieved from the forensic medicine examination reports, the hospital information management system records, and radiological image archives. A record was made for each patient of their age and gender, together with detailed accident-related characteristics. In this context, evaluations were made of the vehicle type involved in the accident (motor car, motorcycle, bicycle, etc.), whether the person injured was a pedestrian or the driver or passenger (within vehicle/outside the vehicle), and the direction of the impact (from the front, side, or back). Information regarding the accident type, role in the accident (driver, passenger, or pedestrian), and impact direction was obtained from official traffic accident reports and judicial investigation records included in the forensic case files. Variables such as helmet use and airbag deployment were evaluated only within the relevant accident subgroups; therefore, denominators differ according to the applicability of each variable.

Radiological evaluation was performed using computed tomography (CT) and/or panoramic radiography images obtained from the hospital imaging archive system. In cases where imaging had been performed at external healthcare institutions, radiological records were accessed through the national electronic health record system (e-Nabız).

CT imaging was available in all cases (94/94), and panoramic radiography was additionally available in a subset of patients (26/94). The choice of imaging modality was based on clinical indication and data availability.

Radiological findings were primarily based on official reports prepared by radiology specialists and, when applicable, by relevant clinical departments such as plastic surgery and neurosurgery. In addition, all available images were reviewed by the authors, both of whom are forensic medicine specialists who are experienced in trauma assessment.

Fracture localization was determined according to standard anatomical mandibular regions, including the symphysis, parasymphysis, corpus, angle, ramus, condyle, and coronoid process. Evaluations of the displacement status (displaced/non-displaced) and whether they were unilateral or bilateral were also made.

In 12 cases, fracture localization could not be reliably determined due to insufficient image quality or inconsistencies between the available radiological images and the corresponding radiology reports. To avoid potential misclassification of fracture location, these cases were excluded from the localization analyses.

The anatomic classification of mandibular fractures used in this study followed widely accepted clinical frameworks for mandibular fracture localization [14]. Fracture displacement was determined based on radiological evaluation, in accordance with radiology reports. A fracture was considered displaced when there was visible cortical discontinuity with separation of bone segments on imaging and/or when displacement was explicitly reported in the radiology report. Mandibular fractures were recorded according to their anatomic location. Fractures occurring on one side were classified as unilateral, while fractures involving both sides were classified as bilateral.

Whether or not there were any facial bone or skull fractures accompanying the mandibular fracture was also recorded. Injuries occurring in other bodily parts such as the thorax, abdomen, pelvis, and extremities were evaluated together with other accompanying bone fractures. Thus, we analyzed whether the mandibular fractures were isolated or occurred as part of multiple traumas.

### Statistical Analysis

Data obtained in the study were analyzed statistically, using SPSS vn. 19.0 software (Statistical Package for the Social Sciences, IBM Corp., Armonk, NY, USA). Conformity of the data to the normal distribution was assessed with the Shapiro–Wilk test. Descriptive statistics were presented as mean ± standard deviation (SD) values for continuous variables and as number (*n*) and percentage (%) for categorical data. The Pearson Chi-square test was used in the examination of the relationships between categorical variables. To control the error share in paired comparisons of multiple groups, the Bonferroni correction was applied. The Bonferroni correction was applied to multiple comparisons within each set of fracture-site-specific analyses. When more than 20% of the expected cell counts were less than five, Fisher’s exact test was used. When Fisher’s exact test could not be calculated (in 2xR Tables), *p*-values were estimated using the Monte Carlo method. In the comparisons of two independent groups of continuous data, the Independent Samples *t*-test was used. A value of *p* < 0.05 was accepted as the level of statistical significance in all the analyses.

This study was designed and reported in accordance with the STROBE guidelines for observational studies [15].

## 3. Results

Mandibular trauma occurred after a traffic accident in 94 individuals, comprising 68.1% males and 31.9% females with a mean age of 29.27 ± 14.34 years. The traffic accidents were most often (47.9%) defined as being inside the vehicle traffic accident (IVTA), and most of the cases were the driver (43.6%) or a passenger (39.4%). The type of vehicle was recorded most often as passenger transport (45.7%) and motorcycle (31.9%), and the vast majority of the accidents were in the form of impact from the front (74.5%). The other characteristics of the accidents are presented in Table 1.

Of the 94 cases included in the study, fracture localization could not be determined in 12 cases, due to insufficient radiological information. Therefore, analyses involving fracture localization were conducted on 82 cases, whereas demographic and accident-related analyses included all 94 cases. Comparisons between cases with determined (*n* = 82) and undetermined (*n* = 12) fracture localization revealed no statistically significant differences across variables including age, sex, accident type, injured person, vehicle type, direction of impact, fracture orientation, fracture form and fracture type (*p* > 0.05). Of the total mandibular fractures, 36.2% were on the left side, 98.9% were closed, and 52.1% were displaced. The data of the number and localization of the mandibular fractures were obtained from 87.2% (*n* = 82) of the records. Of these patients, a single fracture occurred in the mandible of 54.9%. The mandibular fractures were seen most frequently in the ramus (32.9%) and the condyle (32.9%). In all the patients, fracture of other facial bones was seen at the rate of 23.4% and of cranial bones at 20.2% (Table 2).

Condylar fracture was determined in 32.9% of the cases. No statistically significant correlation was determined between the presence of condylar fracture and age (*p* = 0.488). No statistically significant correlation was determined between the development of condylar fracture and gender, accident type, characteristics of the injured person, type of vehicle, direction of impact, and fracture orientation (*p* > 0.05). In cases with abdominal injury, the frequency of condyle fracture was found to be significantly lower (*p* = 0.004) (Table 3).

Ramus fracture was determined in 32.9% of the cases. No statistically significant correlation was determined between the presence of ramus fracture and age (*p* = 0.580). The frequency of ramus fracture was significantly higher in males than in females (*p* = 0.008), and in drivers than in passengers (*p* = 0.033). No statistically significant correlation was determined between the development of ramus fracture and type of accident, type of vehicle, and direction of impact (*p* > 0.05). In cases with accompanying facial bone fracture, skull fracture, and abdominal injury, the frequency of ramus fracture was found to be significantly higher (*p* = 0.049, *p* = 0.002, *p* = 0.025, respectively) (Table 4).

An angle fracture was determined in 17.1% of the cases. No statistically significant correlation was determined between the presence of angle fracture and age (*p* = 0.618). No statistically significant correlation was determined between the development of angle fracture and gender, accident type, characteristics of the injured person, type of vehicle, direction of impact, fracture orientation, and accompanying injuries (*p* > 0.05) (Table 5).

Corpus fracture was determined in 30.5% of the cases. No statistically significant correlation was determined between the presence of corpus fracture and age (*p* = 0.068). When evaluated according to the type of traffic accident, corpus fracture was observed significantly more frequently in IVTAs than in MAs (*p* = 0.005). When the type of vehicle was examined, the frequency of corpus fracture development was found to be significantly higher in passenger transport accidents than in those involving motorcycles (*p* = 0.004). In cases with thoracic spine and abdominal injuries, corpus fracture was seen at a significantly higher rate than in cases without those accompanying injuries (*p* = 0.026, *p* = 0.018, respectively). No statistically significant correlation was determined between the development of corpus fracture and gender, characteristics of the injured person, direction of impact, and fracture orientation (*p* > 0.05) (Table 6).

Symphysis and parasymphysis fractures were determined in 30.5% of the cases. No statistically significant correlation was determined between the presence of symphysis and parasymphysis fracture and age (*p* = 0.569). No statistically significant correlation was determined between the development of symphysis and parasymphysis fracture and gender, accident type, characteristics of the injured person, type of vehicle, direction of impact, and accompanying injuries (*p* > 0.05). The frequency of symphysis and parasymphysis fracture was seen to be significantly higher in bilateral mandibular fractures (*p* = 0.030) (Table 7).

A single mandibular fracture was determined in 54.9% of cases: two different foci of the fracture in 42.7%, and three in 2.4%. No statistically significant correlation was determined between the number of mandibular fractures and age. No statistically significant correlation was determined between the number of mandibular fractures and gender, accident type, characteristics of the injured person, type of vehicle, direction of impact, and accompanying injuries (*p* > 0.05). The frequency of multiple fractures was found to be significantly higher in bilateral mandibular fractures (*p* < 0.001) (Table 8).

A displaced fracture was determined in 52.1% of the cases with mandibular fracture. When evaluated according to fracture orientation, the frequency of non-displaced fracture was found to be significantly higher in mandibular fractures with right-side localization than in bilateral fractures (*p* = 0.002). No statistically significant correlation was determined between fracture type (displaced/non-displaced) and gender, accident type, characteristics of the injured person, type of vehicle, direction of impact, and accompanying injuries (*p* > 0.05) (Table 9).

## 4. Discussion

The most noteworthy finding of this study was that, among mandibular fractures due to traffic accidents, ramus region fractures were observed at a relatively higher rate. Ramus fractures are generally rarely reported in the literature and are considered to be relatively resistant to trauma due to the thick cortical structure of this region and the surrounding strong muscle tissue. Therefore, the predominance of ramus fractures in this study may be associated with higher-energy traffic accident mechanisms, where traumatic forces may have been transmitted directly or laterally to the mandible. The evaluation of the relationships between mandibular fracture localization and accident-related factors in this study may provide insights into possible trauma mechanisms from a forensic medicine perspective. In this context, the presence of facial, cranial, and other system injuries observed together with ramus fractures may indicate that mandibular fractures should be considered as part of multiple traumas, rather than as isolated facial injuries, potentially reflecting the severity of the trauma.

The finding that a large proportion of mandibular trauma cases in the present study were in the young adult age group, and that the male gender was predominant, is consistent with the previous literature on the demographic distribution of maxillofacial trauma due to traffic accidents [16,17]. The higher incidence of trauma in males and young adults has been associated with greater exposure to traffic environments and riskier driving behaviors, including higher driving speeds [16,17]. In the present study, the predominance of in-vehicle traffic accidents (IVTAs) and injured drivers may indicate that mandibular injuries are related to in-vehicle dynamics during traffic accidents [16]. These findings may be compatible with possible contact mechanisms involving the steering wheel, dashboard, or other interior structures of the vehicle; however, such interpretations should be considered hypothetical.

When the accident-related mechanical characteristics were evaluated, there was significant predominance of anterior impacts, which may reflect the involvement of relatively higher-energy trauma mechanisms, where the mandible could be directly or indirectly exposed to force transmission. It has been reported that in anterior collisions, more potential contact with the steering wheel, dashboard, and other interior hard surfaces plays an important role in the occurrence of mandibular and mid-face injuries [18,19]. The notable rate of motorcycle accidents and collisions with pedestrians also suggests that mandibular trauma may occur more frequently in situations where protective structures are absent or limited. Despite the high rate of helmet use, the fact that mandibular trauma developed can be explained by the chin region being an area that is not directly protected in the design of most helmets and shows that mandibular fractures are not prevented by the presence of protective equipment alone [20,21,22]. These findings support the need to consider the accident type, direction of the impact, and the role of the individual in the accident together in the evaluation of mandibular trauma from both a clinical and forensic medicine perspective.

The vast majority of the mandibular fractures in this study were closed and displaced, suggesting that the trauma sustained in traffic accidents may be high-energy in character. It is noteworthy that fractures of the ramus and condyle were determined at equally high rates. When it is considered that ramus fractures have been rarely reported in the literature [23,24], this finding may indicate that the traumatic force could be transmitted to the mandible directly or in a lateral direction. Although the ramus region is accepted as a segment that is resistant to trauma because of the thick cortical structure and surrounding strong muscle tissue, the development of fracture may be considered a potential indicator of the trauma severity [23,24]. The determination of facial, cranial, and other system injuries accompanying mandibular fractures at substantial rates may suggest that the mandibular fractures in this study were usually not an isolated injury but a part of multiple traumas.

A condylar fracture was determined in approximately one-third of the current study cases, but no statistically significant correlation was observed between the presence of condyle fracture and the variables of age, gender, accident type, location of the injured person in the accident, type of vehicle, and direction of impact. This finding suggests that rather than specific demographic- or accident-related factors, condylar fractures mostly develop as a result of indirect forces transmitted along the mandible. It has been reported in the literature that condyle fractures emerge with the transmission of force along the condyle following impact, especially on the tip of the chin or mandible corpus, and therefore can be seen independently of trauma type or direction [25,26,27]. It was notable that condyle fracture was not determined in the current study cases with abdominal injury, which can be explained by a decrease in the force transferred to the face region, as the traumatic energy was absorbed in different areas of the body. When evaluated overall, it can be understood that the trauma biomechanics is the basic factor determining the occurrence of condyle fracture, and isolated demographic or accident-specific variables provide a limited explanation. Thus, the fundamental determining component of these fractures is the biomechanics of the trauma.

In this study, ramus fractures were determined at an above-normal frequency within the mandibular fractures, and there were found to be significant correlations of this fracture type with the male gender and being the driver. In the literature, ramus fractures have been reported less often than condyle and parasymphysis region fractures, but it has been stated that with the effect of high-energy trauma, the lateral segments of the mandible can be fractured [25,28,29]. Especially in the current study cases, who were in the driver position, ramus fractures were seen more often, which may have been associated with the transfer to posterior–lateral segments of the mandible of the high-energy forces transmitted in an anterior or lateral direction in IVTAs. This finding may suggest that not only anatomic resistance properties, but also the mechanical dynamics of the accident and the position of the individual in the accident may play a role in the formation of ramus fractures. Furthermore, the fact that significant correlations were found between ramus fracture and the presence of concomitant facial bone fractures, skull fractures, and abdominal injuries shows that ramus fractures are not usually isolated mandibular injuries but emerge as a part of high-energy polytrauma conditions. In the literature, maxillofacial fractures have been reported to be often seen together with systemic injuries, especially in serious traffic accidents [13,24]. Therefore, in cases determined with ramus fractures, comprehensive evaluation of accompanying injuries together with the severity and direction of the trauma may be of great importance, both clinically and forensically.

Although angulus fractures were determined in this study, no statistically significant correlation was seen between the presence of angulus fracture and age, gender, type of accident, position of the injured individual in the accident, type of vehicle, direction of impact, and accompanying injuries. This implies that rather than specific demographic characteristics or a single accident scenario, angulus fractures can occur together with the effect of the amount and direction of force transmitted to the mandible and individual anatomic/functional factors (e.g., the presence of a third molar tooth, bone density, and local stress distribution). It has been reported in the literature that the angulus region is one of the segments of the mandible that is prone to fracture, and local structural properties in particular could play a role in fracture formation, so the fact that no significant correlation was determined in the current study can be evaluated as being compatible with the multifactorial structure of this type of fracture [30]. Therefore, rather than focusing on a single risk factor in the evaluation of angulus fractures, it would be more appropriate to examine the trauma biomechanics, together with the individual predisposing characteristics.

Corpus fractures were determined in the current study, but no statistically significant relationship was seen between the presence of corpus fracture and age and gender. However, a higher frequency of corpus fracture was seen in IVTAs than in motorcycle accidents, and as even higher rates were determined in passenger vehicle accidents, this suggested that corpus fractures could be associated more with contact mechanisms within the vehicle. As a result of striking the dashboard, steering wheel, and similar hard surfaces, the forces directly transferred to the mandibular corpus could be determinant in this fracture pattern. It has been reported in the literature that impact dynamics and the use of protective equipment could affect facial fracture patterns, and mandibular fractures constitute an important trauma sub-group in motor vehicle accidents [19]. In addition, corpus fracture was determined more frequently in the current study cases with thoracic spine and abdominal injuries, suggesting that rather than an isolated facial injury, the majority of this type of fracture occur within multi-trauma conditions resulting from high-energy trauma. This finding is compatible with the results of extensive series that have emphasized the need for interpretation by evaluating the mechanism of mandibular fractures together with trauma energy and concomitant system injuries [13].

Although symphysis and parasymphysis fractures were determined in the current study, no statistically significant correlation was seen between the presence of these fractures and age, gender, accident type, position of the injured person in the accident, type of vehicle, impact direction, and accompanying injuries. However, when evaluated in terms of the fracture orientation, it was noticeable that the frequency of fractures in the symphysis/parasymphysis region was significantly higher in cases with bilateral mandibular fractures. This finding may suggest that when the traumatic force is transmitted along the mandible and stress is concentrated in the midline, the symphysis and parasymphysis region may be a segment that is more predisposed to fracture. The symphysis/parasymphysis region has been reported to be one of the areas of the mandible that is most frequently fractured, and especially in impacts directed at the anterior mandible, the accumulation of stress in this area is increased [2,9]. Therefore, it has been concluded that rather than evaluation as a single accident parameter, it would be more appropriate to examine symphysis/parasymphysis fractures together with the trauma biomechanics and the fracture combination pattern.

A single fracture line was determined in more than half of the current study cases, whereas two or more fracture foci were seen in a considerable group, demonstrating that rather than fracture of the mandible from a single point during trauma, multifocal damage could develop due to the force distribution along the jaw. As multiple fractures were seen, especially in a bilateral fracture pattern, this implies that the impact not only created a local effect but that simultaneous fractures can emerge in different segments with the transmission of stress along the mandible. Biomechanical approaches explaining the mechanism of mandibular fractures and extensive case series have emphasized that there is often a relationship of multiple fractures with high energy trauma and the widespread distribution of the force on the mandible [10,13,29]. Therefore, in cases determined with multiple mandibular fractures, not only treatment for the fracture line but a holistic evaluation in respect of trauma severity, extent of the fracture pattern, and possible accompanying injuries, is important in terms of both clinical management and forensic medicine reporting.

More than half of the mandibular fractures in this study were displaced, demonstrating that separation between fragments can be often seen together with the effect of high energy in mandibular trauma due to traffic accidents. In terms of the fracture orientation, non-displaced fractures were observed more often in fractures that were determined on the right side, which could indicate that they remained stable without any evident change in the fracture line and the trauma force was absorbed in a more limited segment in some cases. In the literature, it has been reported that the degree of displacement of mandibular fractures is associated with biomechanical factors such as the trauma severity, the direction of force transmission, and muscle traction, and mandibular fractures in traffic accidents could show more complex patterns [10,13,31]. Therefore, in the forensic medicine evaluation of displaced fractures, it would be appropriate to treat the severity of the trauma as a finding that increases the risk of potential functional loss, and to systematically examine accompanying injuries.

This study had some limitations that should be considered when interpreting the findings. First, the cases were identified through forensic medicine evaluations, rather than through a clinical trauma or maxillofacial surgery admission cohort. Therefore, the study population may represent a subgroup of traffic accident patients referred for medico-legal assessment. For this reason, the findings should be interpreted within the context of a forensic medicine referral population, rather than the entire population of mandibular fractures resulting from traffic accidents. In addition, the study did not include direct measures of crash severity, such as vehicle speed, contact points, restraint use, or standardized injury-severity scoring systems. Information regarding accident characteristics, including role in the accident and impact direction, was obtained from traffic accident reports and judicial investigation records, which may have been subject to reporting limitations or incomplete documentation. Therefore, the mechanistic interpretations regarding impact direction or trauma energy should be interpreted cautiously and considered as plausible hypotheses, rather than definitive causal conclusions.

These cases comprised individuals who presented to a forensic medicine unit for medico-legal evaluation following traffic accidents, rather than from a consecutive clinical trauma cohort. In this setting, cases requiring medico-legal documentation of injuries may be more likely to be evaluated, which could lead to an overrepresentation of more severe injuries. Therefore, the observed distribution of mandibular fracture patterns should be interpreted with caution and may not fully reflect the distribution seen in the general population of traffic accident patients.

## 5. Conclusions

The results of this study suggest that mandibular fractures resulting from traffic accidents may not remain limited to a single fracture line, and certain fracture patterns, especially multiple mandibular fractures and accompanying systemic injuries, can be seen together. The findings obtained in the study present important information about the localization of mandibular fractures, fracture characteristics (e.g., displacement status and the presence of multiple fractures), and the biomechanics and severity of the trauma, and show that a systematic approach is necessary in forensic medicine evaluation.

In this context, rather than focusing only on the facial region in cases determined to have mandibular fracture, a holistic investigation of intracranial events and accompanying injuries in other body areas is of great importance in respect to correct evaluation of the trauma severity, objectivity of the forensic reporting, and effective clinical management.

## Figures and Tables

**Table 1:** Characteristics of the accidents.

		Number (*n*)	Percentage (%)
Accident Type *	IVTA	45	47.9
	OVTA **	19	20.2
	MA	30	31.9
Injured Person	Passenger	37	39.4
	Driver	41	43.6
	Pedestrian	16	17.0
Vehicle type	Passenger transport	43	45.7
	Bicycle	3	3.2
	Large vehicle ***	3	3.2
	Motorcycle	30	31.9
	Pedestrian ****	15	16.0
Use of helmet (*n* = motorcycle accidents)	No	1	3.3
	Yes	29	96.7
Airbag (*n* = in-vehicle occupants)	Not opened	45	100.0
	Opened	0	0.0
Direction of Impact	From the back	5	5.3
	From the front	70	74.5
	From the right side	10	10.6
	From the left side	9	9.6

* IVTA: inside the vehicle traffic accident, OVTA: outside the vehicle traffic accident, and MA: motorcycle accident. ** The pedestrian category represents injuries sustained in traffic accidents outside the vehicle. *** The large vehicle category includes lorries, pick-up trucks, and buses. **** “Vehicle type” represents the type of traffic participant involved in the accident; therefore, pedestrians are included within this category.

**Table 2:** Fracture characteristics and accompanying injuries.

		Number (*n*)	Percentage (%)
Fracture orientation (*n* = 94)	Bilateral	32	34.0
	Midline	1	1.1
	Right	27	28.7
	Left	34	36.2
Fracture Form (*n* = 94)	Closed	93	98.9
	Open	1	1.1
Fracture type (*n* = 94)	Displaced	49	52.1
	Non-displaced	45	47.9
Condyle fracture (*n* = 82) *	Present	27	32.9
Coronoid fracture (*n* = 82) *	Present	1	1.2
Ramus fracture (*n* = 82) *	Present	27	32.9
Angle fracture (*n* = 82) *	Present	14	17.1
Corpus fracture (*n* = 82) *	Present	25	30.5
Parasymphysis fracture (*n* = 82) *	Present	19	23.2
Symphysis fracture (*n* = 82) *	Present	8	9.8
Number of mandibular fractures (*n* = 82) *	One	45	54.9
	Two	35	42.7
	Three	2	2.4
Concomitant facial fracture (*n* = 94)	Present	22	23.4
Skull fracture (*n* = 94)	Present	19	20.2
Intracranial event (*n* = 94)	Present	11	11.7
Cervical spine injury (*n* = 94)	Present	5	5.3
Thoracic spine injury (*n* = 94)	Present	16	17.0
Abdominal injury (*n* = 94)	Present	14	14.9
Extremity injury (*n* = 94)	Present	34	36.2

* In 12 of the total 94 cases, the presence of fracture was determined on radiological images, but the localization could not be clearly distinguished. Therefore, the analyses of the number and localization of the fractures was conducted on 82 cases for which the data could be definitely confirmed.

**Table 3:** Condylar region fractures and associated factors.

		Condyle Fracture				
		Absent		Present		
		*n*	%	*n*	%	*p*
Gender	Female	17	68.0	8	32.0	0.906
	Male	38	66.7	19	33.3	
Accident type	IVTA	29	74.4	10	25.6	0.121
	OVTA	12	75.0	4	25.0	
	MA	14	51.9	13	48.1	
Injured person	Passenger	21	67.7	10	32.3	0.702 **
	Driver	24	63.2	14	36.8	
	Pedestrian	10	76.9	3	23.1	
Vehicle type	Passenger transport	28	73.7	10	26.3	0.313 **
	Bicycle	2	66.7	1	33.3	
	Large vehicle	2	100.0	0	0.0	
	Motorcycle	14	51.9	13	48.1	
	Pedestrian	9	75.0	3	25.0	
Direction of impact	From the back	4	80.0	1	20.0	0.677 **
	From the front	39	62.9	23	37.1	
	From the right side	6	85.7	1	14.3	
	From the left side	6	75.0	2	25.0	
Fracture orientation	Bilateral	15	53.6	13	46.4	0.269 **
	Midline	1	100.0	0	0.0	
	Right	15	71.4	6	28.6	
	Left	24	75.0	8	25.0	
Facial fracture	Absent	43	66.2	22	33.8	1.000
	Present	12	70.6	5	29.4	
Skull fracture	Absent	43	66.2	22	33.8	1.000
	Present	12	70.6	5	29.4	
Intracranial event	Absent	48	67.6	23	32.4	1.000 *
	Present	7	63.6	4	36.4	
Cervical spine injury	Absent	51	66.2	26	33.8	1.000 *
	Present	4	80.0	1	20.0	
Thoracic spine injury	Absent	44	64.7	24	35.3	0.369 *
	Present	11	78.6	3	21.4	
Abdominal injury	Absent	42	60.9	27	39.1	0.004 *
	Present	13	100.0	0	0.0	
Extremity injury	Absent	34	61.8	21	38.2	0.148
	Present	21	77.8	6	22.2	

Pearson Chi-square test was used. * Fisher’s exact test was used. ** Monte Carlo method was used.

**Table 4:** Ramus region fractures and associated factors.

		Ramus Fracture				
		Absent		Present		
		*n*	%	*n*	%	*p*
Gender	Female	22	88.0	3	12.0	0.008
	Male	33	57.9	24	42.1	
Accident type	IVTA	28	71.8	11	28.2	0.560
	OVTA	11	68.8	5	31.3	
	MA	16	59.3	11	40.7	
Injured person	Passenger ^a^	26	83.9	5	16.1	0.031 **
	Driver ^b^	21	55.3	17	44.7	
	Pedestrian ^a,b^	8	61.5	5	38.5	
Vehicle type	Passenger transport	26	68.4	12	31.6	0.690 **
	Bicycle	3	100.0	0	0.0	
	Large vehicle	2	100.0	0	0.0	
	Motorcycle	16	59.3	11	40.7	
	Pedestrian	8	66.7	4	33.3	
Direction of impact	From the back	3	60.0	2	40.0	1.000 **
	From the front	41	66.1	21	33.9	
	From the right side	5	71.4	2	28.6	
	From the left side	6	75.0	2	25.0	
Fracture orientation	Bilateral	19	67.9	9	32.1	1.000 **
	Midline	1	100.0	0	0.0	
	Right	14	66.7	7	33.3	
	Left	21	65.6	11	34.4	
Facial fracture	Absent	47	72.3	18	27.7	0.049
	Present	8	47.1	9	52.9	
Skull fracture	Absent	49	75.4	16	24.6	0.002
	Present	6	35.3	11	64.7	
Intracranial event	Absent	49	69.0	22	31.0	0.491 *
	Present	6	54.5	5	45.5	
Cervical spine injury	Absent	53	68.8	24	31.2	0.325 *
	Present	2	40.0	3	60.0	
Thoracic spine injury	Absent	45	66.2	23	33.8	1.000 *
	Present	10	71.4	4	28.6	
Abdominal injury	Absent	50	72.5	19	27.5	0.025 *
	Present	5	38.5	8	61.5	
Extremity injury	Absent	39	70.9	16	29.1	0.291
	Present	16	59.3	11	40.7	

Pearson Chi-square test was used. * Fisher’s exact test was used. ** Monte Carlo method was used. Different superscripts (^a,b^) indicate Bonferroni-adjusted significant differences, shown only for variables with >2 groups and significant χ^2^ tests.

**Table 5:** Angle region fractures and associated factors.

		Angle Fracture				
		Absent		Present		
		*n*	%	*n*	%	*p* *
Gender	Female	19	76.0	6	24.0	0.341
	Male	49	86.0	8	14.0	
Accident type	IVTA	32	82.1	7	17.9	1.000 **
	OVTA	13	81.3	3	18.8	
	MA	23	85.2	4	14.8	
Injured person	Passenger	24	77.4	7	22.6	0.482 **
	Driver	32	84.2	6	15.8	
	Pedestrian	12	92.3	1	7.7	
Vehicle type	Passenger transport	31	81.6	7	18.4	0.237 **
	Bicycle	1	33.3	2	66.7	
	Large vehicle	2	100.0	0	0.0	
	Motorcycle	23	85.2	4	14.8	
	Pedestrian	11	91.7	1	8.3	
Direction of impact	From the back	4	80.0	1	20.0	0.634 **
	From the front	51	82.3	11	17.7	
	From the right side	7	100.0	0	0.0	
	From the left side	6	75.0	2	25.0	
Fracture orientation	Bilateral	21	75.0	7	25.0	0.527 **
	Midline	1	100.0	0	0.0	
	Right	18	85.7	3	14.3	
	Left	28	87.5	4	12.5	
Facial fracture	Absent	52	80.0	13	20.0	0.280
	Present	16	94.1	1	5.9	
Skull fracture	Absent	52	80.0	13	20.0	0.280
	Present	16	94.1	1	5.9	
Intracranial event	Absent	57	80.3	14	19.7	0.197
	Present	11	100.0	0	0.0	
Cervical spine injury	Absent	63	81.8	14	18.2	0.582
	Present	5	100.0	0	0.0	
Thoracic spine injury	Absent	54	79.4	14	20.6	0.113
	Present	14	100.0	0	0.0	
Abdominal injury	Absent	56	81.2	13	18.8	0.450
	Present	12	92.3	1	7.7	
Extremity injury	Absent	44	80.0	11	20.0	0.369
	Present	24	88.9	3	11.1	

* Fisher’s exact test was used. ** Monte Carlo method was used.

**Table 6:** Corpus region fractures and associated factors.

		Corpus Fracture				
		Absent		Present		
		*n*	%	*n*	%	*p*
Gender	Female	15	60.0	10	40.0	0.215
	Male	42	73.7	15	26.3	
Accident type	IVTA ^a^	22	56.4	17	43.6	0.005 **
	OVTA ^a,b^	10	62.5	6	37.5	
	MA ^b^	25	92.6	2	7.4	
Injured person	Passenger	19	61.3	12	38.7	0.069 **
	Driver	31	81.6	7	18.4	
	Pedestrian	7	53.8	6	46.2	
Vehicle type	Passenger transport ^a^	21	55.3	17	44.7	0.004 **
	Bicycle ^a,b^	3	100.0	0	0.0	
	Large vehicle ^a,b^	1	50.0	1	50.0	
	Motorcycle ^b^	25	92.6	2	7.4	
	Pedestrian ^a,b^	7	58.3	5	41.7	
Direction of impact	From the back	4	80.0	1	20.0	0.091 **
	From the front	46	74.2	16	25.8	
	From the right side	2	28.6	5	71.4	
	From the left side	5	62.5	3	37.5	
Fracture orientation	Bilateral	18	64.3	10	35.7	0.766 **
	Midline	1	100.0	0	0.0	
	Right	14	66.7	7	33.3	
	Left	24	75.0	8	25.0	
Facial fracture	Absent	45	69.2	20	30.8	0.914
	Present	12	70.6	5	29.4	
Skull fracture	Absent	45	69.2	20	30.8	0.914
	Present	12	70.6	5	29.4	
Intracranial event	Absent	48	67.6	23	32.4	0.490 *
	Present	9	81.8	2	18.2	
Cervical spine injury	Absent	53	68.8	24	31.2	1.000 *
	Present	4	80.0	1	20.0	
Thoracic spine injury	Absent	51	75.0	17	25.0	0.026 *
	Present	6	42.9	8	57.1	
Abdominal injury	Absent	52	75.4	17	24.6	0.018 *
	Present	5	38.5	8	61.5	
Extremity injury	Absent	42	76.4	13	23.6	0.054
	Present	15	55.6	12	44.4	

Pearson Chi-square test was used. * Fisher’s exact test was used, ** Monte Carlo method was used. Different superscripts (^a,b^) indicate Bonferroni-adjusted significant differences, shown only for variables with >2 groups and significant χ^2^ tests.

**Table 7:** Symphysis and parasymphysis fractures and associated factors.

		Symphysis and Parasymphysis Fracture				
		Absent		Present		
		*n*	%	*n*	%	*p*
Gender	Female	16	64.0	9	36.0	0.473
	Male	41	71.9	16	28.1	
Accident type	IVTA	26	66.7	13	33.3	0.567 **
	OVTA	13	81.3	3	18.8	
	MA	18	66.7	9	33.3	
Injured person	Passenger	17	54.8	14	45.2	0.088 **
	Driver	30	78.9	8	21.1	
	Pedestrian	10	76.9	3	23.1	
Vehicle type	Passenger transport	26	68.4	12	31.6	0.831 **
	Bicycle	3	100.0	0	0.0	
	Large vehicle	1	50.0	1	50.0	
	Motorcycle	18	66.7	9	33.3	
	Pedestrian	9	75.0	3	25.0	
Direction of impact	From the back	4	80.0	1	20.0	1.000 **
	From the front	42	67.7	20	32.3	
	From the right side	5	71.4	2	28.6	
	From the left side	6	75.0	2	25.0	
Fracture orientation	Bilateral ^a^	15	53.6	13	46.4	0.030 **
	Midline ^a^	0	0.0	1	100.0	
	Right ^a^	18	85.7	3	14.3	
	Left ^a^	24	75.0	8	25.0	
Facial fracture	Absent	45	69.2	20	30.8	0.914
	Present	12	70.6	5	29.4	
Skull fracture	Absent	43	66.2	22	33.8	0.196
	Present	14	82.4	3	17.6	
Intracranial event	Absent	50	70.4	21	29.6	0.729 *
	Present	7	63.6	4	36.4	
Cervical spine injury	Absent	53	68.8	24	31.2	1.000 *
	Present	4	80.0	1	20.0	
Thoracic spine injury	Absent	50	73.5	18	26.5	0.112 *
	Present	7	50.0	7	50.0	
Abdominal injury	Absent	47	68.1	22	31.9	0.745 *
	Present	10	76.9	3	23.1	
Extremity injury	Absent	40	72.7	15	27.3	0.367
	Present	17	63.0	10	37.0	

Pearson Chi-square test was used. * Fisher’s exact test was used. ** Monte Carlo method was used. Different superscripts (^a^) indicate Bonferroni-adjusted significant differences, shown only for variables with >2 groups and significant χ^2^ tests.

**Table 8:** Number of mandibular fractures and associated factors.

		Number of Fractures						
		One		Two		Three		
		*n*	%	*n*	%	*n*	%	*p* *
Gender	Female	13	52.0	12	48.0	0	0.0	0.741
	Male	32	56.1	23	40.4	2	3.5	
Accident type	IVTA	18	46.2	21	53.8	0	0.0	0.137
	OVTA	11	68.8	5	31.3	0	0.0	
	MA	16	59.3	9	33.3	2	7.4	
Injured person	Passenger	13	41.9	17	54.8	1	3.2	0.374
	Driver	24	63.2	13	34.2	1	2.6	
	Pedestrian	8	61.5	5	38.5	0	0.0	
Vehicle type	Passenger transport	16	42.1	22	57.9	0	0.0	0.105
	Bicycle	3	100.0	0	0.0	0	0.0	
	Large vehicle	2	100.0	0	0.0	0	0.0	
	Motorcycle	16	59.3	9	33.3	2	7.4	
	Pedestrian	8	66.7	4	33.3	0	0.0	
Direction of impact	From the back	4	80.0	1	20.0	0	0.0	0.240
	From the front	31	50.0	30	48.4	1	1.6	
	From the right side	5	71.4	1	14.3	1	14.3	
	From the left side	5	62.5	3	37.5	0	0.0	
Fracture orientation	Bilateral	6 ^a^	21.4	20 ^a^	71.4	2 ^a^	7.1	<0.001
	Midline	1 ^a,b^	100.0	0 ^a,b^	0.0	0 ^a^	0.0	
	Right	15 ^b^	71.4	6 ^b^	28.6	0 ^a^	0.0	
	Left	23 ^b^	71.9	9 ^b^	28.1	0 ^a^	0.0	
Facial fracture	Absent	35	53.8	29	44.6	1	1.5	0.473
	Present	10	58.8	6	35.3	1	5.9	
Skull fracture	Absent	35	53.8	29	44.6	1	1.5	0.473
	Present	10	58.8	6	35.3	1	5.9	
Intracranial event	Absent	37	52.1	33	46.5	1	1.4	0.075
	Present	8	72.7	2	18.2	1	9.1	
Cervical spine injury	Absent	42	54.5	33	42.9	2	2.6	1.000
	Present	3	60.0	2	40.0	0	0.0	
Thoracic spine injury	Absent	39	57.4	28	41.2	1	1.5	0.282
	Present	6	42.9	7	50.0	1	7.1	
Abdominal injury	Absent	39	56.5	28	40.6	2	2.9	0.678
	Present	6	46.2	7	53.8	0	0.0	
Extremity injury	Absent	33	60.0	21	38.2	1	1.8	0.313
	Present	12	44.4	14	51.9	1	3.7	

* Monte Carlo method was used. Different superscripts in the same column indicate statistically significant differences between fracture orientation based on post hoc pairwise comparisons with Bonferroni adjustment. Pairwise comparisons for the “2 vs. 3 fractures” category could not be computed for the Midline, Right, and Left groups, due to zero cell frequencies and lack of variance in the number of fractures category. Different superscripts (^a,b^) indicate Bonferroni-adjusted significant differences, shown only for variables with >2 groups and significant χ^2^ tests.

**Table 9:** Fracture type and associated factors.

		Fracture Type				
		Displaced		Non-Displaced		
		*n*	%	*n*	%	*p*
Gender	Female	14	46.7	16	53.3	0.468
	Male	35	54.7	29	45.3	
Accident type	IVTA	24	53.3	21	46.7	0.898
	OVTA	9	47.4	10	52.6	
	MA	16	53.3	14	46.7	
Injured person	Passenger	19	51.4	18	48.6	0.936
	Driver	21	51.2	20	48.8	
	Pedestrian	9	56.3	7	43.8	
Vehicle type	Passenger transport	24	55.8	19	44.2	0.498 **
	Bicycle	0	0.0	3	100.0	
	Large vehicle	1	33.3	2	66.7	
	Motorcycle	16	53.3	14	46.7	
	Pedestrian	8	53.3	7	46.7	
Direction of impact	From the back	3	60.0	2	40.0	0.285 **
	From the front	39	55.7	31	44.3	
	From the right side	5	50.0	5	50.0	
	From the left side	2	22.2	7	77.8	
Fracture orientation	Bilateral ^a^	24	75.0	8	25.0	0.002 **
	Midline ^a,b^	0	0.0	1	100.0	
	Right ^a,b^	8	29.6	19	70.4	
	Left ^a^	17	50.0	17	50.0	
Facial fracture	Absent	34	47.2	38	52.8	0.085
	Present	15	68.2	7	31.8	
Skull fracture	Absent	40	53.3	35	46.7	0.642
	Present	9	47.4	10	52.6	
Intracranial event	Absent	44	53.0	39	47.0	0.637
	Present	5	45.5	6	54.5	
Cervical spine injury	Absent	46	51.7	43	48.3	1.000 *
	Present	3	60.0	2	40.0	
Thoracic spine injury	Absent	42	53.8	36	46.2	0.461
	Present	7	43.8	9	56.3	
Abdominal injury	Absent	44	55.0	36	45.0	0.183
	Present	5	35.7	9	64.3	
Extremity injury	Absent	33	55.0	27	45.0	0.459
	Present	16	47.1	18	52.9	

Pearson Chi-square test was used. * Fisher’s exact test was used. ** Monte Carlo method was used Different superscripts (^a,b^) indicate Bonferroni-adjusted significant differences, shown only for variables with >2 groups and significant χ^2^ tests.

## Data Availability

The datasets used and/or analyzed during the current study are not publicly available, due to ethical and legal restrictions related to forensic medical records, but may be made available from the corresponding author upon reasonable request and subject to institutional approval.
